# Genotypic characterization of cashew (*Anacardium occidentale* L.) clones using agro‐morphological traits

**DOI:** 10.1002/pei3.10034

**Published:** 2020-12-02

**Authors:** Paul K. K. Adu‐Gyamfi, Abraham Akpertey, Michael Barnnor, Atta Ofori, Francis Padi

**Affiliations:** ^1^ Cocoa Research Institute of Ghana Accra Ghana

**Keywords:** *Anarcadium*, combining ability, heritability, trunk cross‐sectional area

## Abstract

High cropping efficiency implies that high yields are obtained from reasonably sized trees. We studied the general and specific combining ability (GCA and SCA) of selected cashew clones of Brazilian (A), Beninese (BE), and Ghanaian (SG) background for cropping efficiency and nut weight in the early years of bearing. Using North Carolina II mating design, four clones were crossed as males to three best clones recommended for farmers. The 12 F_1_ progenies were evaluated in the field at Wenchi (2012–2018) for increase in trunk cross‐sectional area at the vegetative (TCSAv) and reproductive (TCSAr) stages, canopy spread in the east‐west (CSew) and north‐south (CSns) directions, nut yield (NY), nut weight (NW), and cropping efficiency (CE) using a randomized complete block design with three replications. Cropping efficiencies were in the range of 30.8–67.4 g/cm^2^/year while nut weight and nut yield varied from 5.9 to 10.5 g/year and 477.8 to 939.4 kg ha^‐1^ year^‐1^ in the fourth to sixth years after planting, respectively. The Beninese progenies outperformed the Brazilian progenies for cropping efficiency. GCA effects were more important than SCA effects. Narrow‐sense heritability ranged from 0.47 (CE) to 0.80 (NW). Canopy spread in the north‐south direction correlated (*rg* = 0.98; *p ≤ *.001) strongly with cropping efficiency at the genotypic level. Among males, BE203 showed positive GCA effects for cropping efficiency, TCSAv, and nut yield, whereas A2 and SG273 showed positive GCA effects for nut weight. Among females, SG287 showed negative GCA effects for TCSAr. Our study provides evidence that, cashew tree size and nut quality are under genetic control and the identified clones represent a suitable genetic resource pool to increase productivity.

## 1 INTRODUCTION

Cashew (*Anacardium occidentale* L.) is a perennial tree crop and a member of the family *Anacardiaceae*. The center of origin is north‐eastern Brazil but was introduced to West Africa by the early Portuguese settlers in the 16th century (Abdul & Peter, 2010; Mitchell & Mori, 1987). The crop is valued for their nuts and apples and has been a major foreign exchange earner for many developing countries in the tropical and subtropical regions of the world (Oliveira, 2008b). Monteiro et al. (2017) reported that about 45% of the world's cashew production comes from West Africa, with Ivory Coast, Ghana, and Nigeria being major producers. The potential of cashew in alleviating poverty and boosting rural development has been highly emphasized (Dendena & Corsi, 2014; Ingram et al., 2015; Wongnaa & Awunyo‐Vitor, 2013). However, low nut yield and nut weight limit the productivity (Adu‐Gyamfi, Dadzie, et al., 2019; Dadzie et al., 2014). The situation is even made worse by the recent awarenes that nut weight is the major criterion that determines the market value of raw cashew nuts in the cashew global trade. Although the low productivity could be partly attributed to pest and diseases, the high global demand for cashew nut from the ever‐increasing world population, coupled with farmers request for varieties that provide high early yield per unit area with "jumbo" nuts that can earn premium price suggest the development of new varieties with high genetic potential for higher nut yield per unit area and improved nut quality.

Cropping efficiency, which is defined as the ratio of the cumulative yield over a period of time to increase in trunk cross‐sectional area over the same period (Daymond et al., 2002), is analogous to harvest efficiency in annual crops (Pang, 2006). High cropping efficiency in general term relates to a significant reduction in vegetative vigor and size during production while the adverse effects of high seedling vigor on crop management are minimized (Daymond et al., 2002; Padi et al., 2012). According to Padi et al. (2017), it implies that high yields are obtained in reasonably sized trees allowing crop management practices such as pruning, harvesting, and pesticide application to be possible over the life cycle of the crop. In orchard crops, which includes cashew, a combination of small tree size and high yield efficiency indicates the potential for high nut yield per unit area (Larsen et al., 1992). The trait is being used by plant breeders to increase productivity through high‐density planting.

Cashew has been grouped into common and dwarf cultivars based on genetic variability. The common types are widely cultivated with heights ranging from 5 to 8 m and canopy diameter reaching 20 m, whereas the dwarf types are less than 4 m in height, having a homogeneous canopy with diameters less than the common type (Oliveira, 2008a). These imply that the potential size of cashew trees is under genetic control and varieties with different tree sizes can be developed by breeding.

Over the years, a number of genetically diverse cashew germplasm accessions have been introduced from the center of origin to various cashew‐growing countries in the West African subregion. In Ghana, the first introductions were from Brazil, Benin, Nigeria, Mozambique, and Tanzania (Dadzie et al., 2014). The introductions from Brazil were dwarf types while those from Benin were common types with high tolerance to hostile environments (Adu‐Gyamfi, Abu Dadzie, et al., 2019).

Recently, the narrow genetic base of cultivated cashew clones particularly in Africa and Asia has been attributed to low cashew productivity (Aliyu, 2012; Archak et al., 2003). To counteract this low productivity, many tree crop improvement strategies have strongly emphasized the introgression of desirable exotic alleles into commercially cultivated local varieties which are adapted to local environmental conditions (Ofori et al., 2014; Padi, Ofori, & Akpertey, 2017). Therefore, the occurrence of the local common cashew clones recommended for farmers together with the exotic precocious Benin and Brazilian dwarf germplasm clones indicate a good opportunity to simultaneously reduce tree vigor/size, improve nut weight and subsequently improve cropping efficiency. However, it is crucial to expand on the limited information regarding the combining abilities of these clones of varied introduction background and the type of gene action governing the inheritance of key economic traits for the current cashew breeding program that focuses on progenies as varieties. Earlier studies on cashew have reported significant effects of both GCA and SCA on nut yield (Cavalcanti et al., 1997), kernel weight, and plant height (Cavalcanti et al., 2007), showing prospects in the improvement of the crop for cropping efficiency and other yield related traits.

In tree crop breeding, extended periods of time in addition to the large amount of resources and labor are required for effective evaluation. Therefore, knowledge of the relationship between traits and parental values in crosses is crucial, as it ensures that effective parental choices are made and genetic breeding progress can be predicted (Ofori et al., 2014a). Over the years, juvenile traits in many tree crop breeding programs have been used as indices for selecting high yielding genotypes in later years (Padi et al., 2012).

The objectives of the present study were to determine (i) the combining abilities of selected Brazilian, Beninese, and Ghanaian cashew clones for vigor, cropping efficiency, and nut weight and (ii) the genotypic and phenotypic correlations between growth and cropping‐efficiency‐related traits.

## 2 MATERIALS AND METHODS

### 2.1 Cashew parental clones, experimental design, and crop management

The plant material used in this experiment involved two exotic and five local parental clones. The exotic parental clones selected for the study were BE203 from Benin and A2 from Brazil while the local (Ghana) parental clones selected for this study were SG273, SG224 ,SG266, SG276, and SG287 (Table 1). Using North Carolina II mating scheme, manual pollinations were carried out with clones BE203, A2, SG224, and SG273 as males and SG266, SG276, and SG287 as females during October 2011–February 2012 to produce 12 F_1_ progenies. On the basis of the origin of male parent, the 12 F_1_ progenies generated were classified into genotype groups: Ghana 1 (SG266 × SG273, SG287 × SG273, and SG276 × SG273), Ghana 2 (SG276 × SG224, SG266 × SG224, and SG287 × SG224), Benin (SG287 × BE203, SG276 × BE203, and SG266 × BE203), and Brazil (SG266 × A2, SG276 × A2, and SG287 × A2) progenies. The Ghana 1 and 2 served as the standard varieties/controls. The male and female parents in this study were selected based on preliminary germplasm evaluation experiment conducted at Wenchi and Bole research stations in Ghana from 2004 to 2012. Selection criteria included vigor, nut yield, and nut weight (Cocoa Research Institute of Ghana Annual reports, 2010). The three female parents were the top three best performing local clones recommended for farmers. These clones were selected from a historical local germplasm collection plot assembled in the mid‐1990s. A description of the characteristics of parental clones utilized is given in Table 1. The experiment was conducted at the Wenchi Agricultural Research Station (N 07° 45. 740′, W 002° 05. 440′) which is situated in the Forest Transitional Agro‐ecological zone. This zone is characterized by a mean annual rainfall of 1,300 mm with mean annual temperature range of 26.1–28.9°C (Lacombe et al., 2012; Owusu & Waylen, 2013). Soil samples were randomly collected from 16 different spots across the experimental site at a depth of 0–30 cm before establishment of the trial. The samples were bulked together and subsamples were taken to the laboratory for analysis. The soils were found to be predominantly lithosols with acidic reaction (pH: 5.1) (Table S1). With the exception of soil organic carbon content, the nitrogen, phosphorus, magnesium, and potassium levels were higher than the recommended levels reported by Dedzoe et al. (2001). The total available phosphorus content was approximately 25% higher than recommended levels. Based on the soil chemical composition, the soils at the experimental site appeared to be fertile. Cashew seedlings were transplanted to the experimental site in June 2012 at a spacing of 10 m × 10 m (100 plants/ha) with 15 plants per plot in a randomized complete block design with three replicates. The standard practices for cashew production in Ghana were duly followed. This included the application of pesticides mainly cyperderm *(active ingredient* – *cypermethrin)* @ 150 ml/ha ) to control insect pest from August to October annually.

**TABLE 1:** Characteristics of cashew clones used as parents for the progenies in a 3 × 4 factorial mating design

Parent	Source of germplasm	Type of cultivar	Nut weight (g/year)
A2	Brazil	Dwarf	High nut weight (˃10 g/year)
BE203	Benin	Common	Moderate nut weight (7 g/year)
SG224	Ghana	Common	Low nut weight (˂7 g/year), (Adu‐Gyamfi et al 2019).
SG273	Ghana	Common	Moderate nut weight (7 g/year) (Adu‐Gyamfi et al 2019).
SG266	Ghana	Common	Low nut weight (˂6 g/year), (Adu‐Gyamfi et al 2019).
SG276	Ghana	Common	Low nut weight (˂7 g/year), (Adu‐Gyamfi et al 2019).
SG287	Ghana	Common	Low nut weight (˂7 g/year), (Adu‐Gyamfi et al 2019).

### 2.2 Measurement of agronomic traits

In general terms, vegetative and reproductive traits were assessed based on the cashew descriptor list of the International Board for Plant Genetic Resources (International Board for Plant Genetics, 1986). The stem diameter of young cashew plants was measured at 15 cm from the soil surface with electronic callipers at 6‐month intervals from December 2012 to December 2014. Data on cumulative nut yield (NY) were estimated from the weight of raw nuts collected per plot throughout the fruiting season from 2014 to 2018. The first‐year records were not analyzed due to large variations, typical of most tree crops. Nut weight (NW) was estimated as the weight of 1 kg of raw cashew nuts produced per plot divided by the number of nuts. Canopy spread (CS) was measured by marking each tree on the side as north, south, east, and west with the help of a GPS device. Measurements were taken per plot from east‐west (CSew) and from the north‐south (CSns) direction at yearly intervals using a tape measure during 2014–2016 cropping season. Vigor was estimated as increase in trunk cross‐sectional area at the vegetative (TCSAv) (2012–2014) and reproductive stages (TCSAr) (2015–2018) using the formula:

$$(\pi d^{2})/4$$

where d is the stem/trunk diameter.

### 2.3 Statistical analysis

For the agronomic data obtained, a two‐stage analysis based on plot‐level values were used in analyses of variance (ANOVA) following tests for normality (based on the plot of residuals). In the first stage, all 12 F_1_ progenies were analyzed to test for significant differences using the average trait values across years, with progenies considered a fixed factor and replicates as random factor, using the GenStat statistical software, version 12 (VSN International Ltd., Hemel Hempstead, UK). In the second stage, progenies were classified into four genotype groups viz, Benin, Brazil, Ghana 1, and Ghana 2 based on the origin of male parent germplasm. The combined means of the three progenies within each genotype group for cropping efficiency and nut weight were determined. At this stage, genotype groups were considered as fixed factor and replicates as random factor. The general and specific combining ability (GCA and SCA) effect, narrow‐sense heritability, additive variance, dominance variance, and environmental variance were estimated using restricted maximum likelihood (REML) methods in AGD‐R (Analysis for Genetic Designs in R) software (Rodríguez et al. 2015).

The GCA and SCA effects were estimated using the following model:

(1) $$Y_{\mathrm{ijk}}=\mu +b_{k}+m_{i}+f_{j}+m_{i}^{*}f_{j}+e_{\mathrm{ijk}}$$

where *Yijk* is the observed value; *µ* is the population mean effect; *b_k_* is the block effect; *m_i_* is the male GCA effect (i = 1; 2; :::;m); *f_j_* is the female GCA effect (j = 1; 2; :::; f); mi ^*^ fj is the SCA effect, and *eijk* is the residual effect. The differences among means were tested by Duncan's Multiple Range Test (DMRT) at the 5% probability level. The genetic and phenotypic correlation coefficients between two traits, i and j, were estimated using METAR‐R statistical package (Alvarado et al., 2018 ). The analysis on vigor (increase in trunk cross‐sectional area at the vegetative stage, TCSAv) utilized the difference between the initial and final recordings taken from December 2012 to December 2014, whereas that on cumulative nut yield and nut weight utilized 4‐year data (2015–2018). Cropping efficiency was estimated as the cumulative nut yield per plot from 2015 to 2018 divided by increase in trunk cross‐sectional area (TCSAr) over the same period. The analysis on canopy spread in the east to west and north to south direction also utilized the difference between initial and final measurements from June 2014 to June 2016. Analysis of the genotype group effects was carried out for only cropping efficiency and nut weight which were the two key traits of interest in this study. The best and worst cashew progenies were concurrently selected based on the overall trait performance using a scale of 1–12 to score for each trait after which the total scores were ranked according to Ofori et al. (2014).

## 3 RESULTS

### 3.1 Analyses of variance

The genotypic effect was highly significant (*p* ˂ .05) for all traits considered in this study. Partitioning of the progeny effect into male, female, and male × female interaction components indicated that the female effect (GCA effects for female) was not significant for any trait except increase in trunk cross‐sectional area at the reproductive stage (TCSAr). Therefore, the contribution of the female effect to the overall variability observed for the other traits among progenies was generally negligible. However, the effect of GCA for males was highly significant for all traits studied except TCSAr and consequently contributed the most to the variation observed among the progenies. On the other hand, the SCA effect was also significant for all traits, with the exception of cropping efficiency and canopy spread in the east‐west (CSew) and north‐south (CSns) directions. The ratio of the additive (GCA) to dominance (SCA) variance was also much higher than unity for most traits and indicated the predominance of GCA effects in controlling most traits. Genotype group effect was also significant for the nut weight and cropping efficiency.

### 3.2 Performance of F_1_ progenies for agronomic traits

**FIGURE 1:**
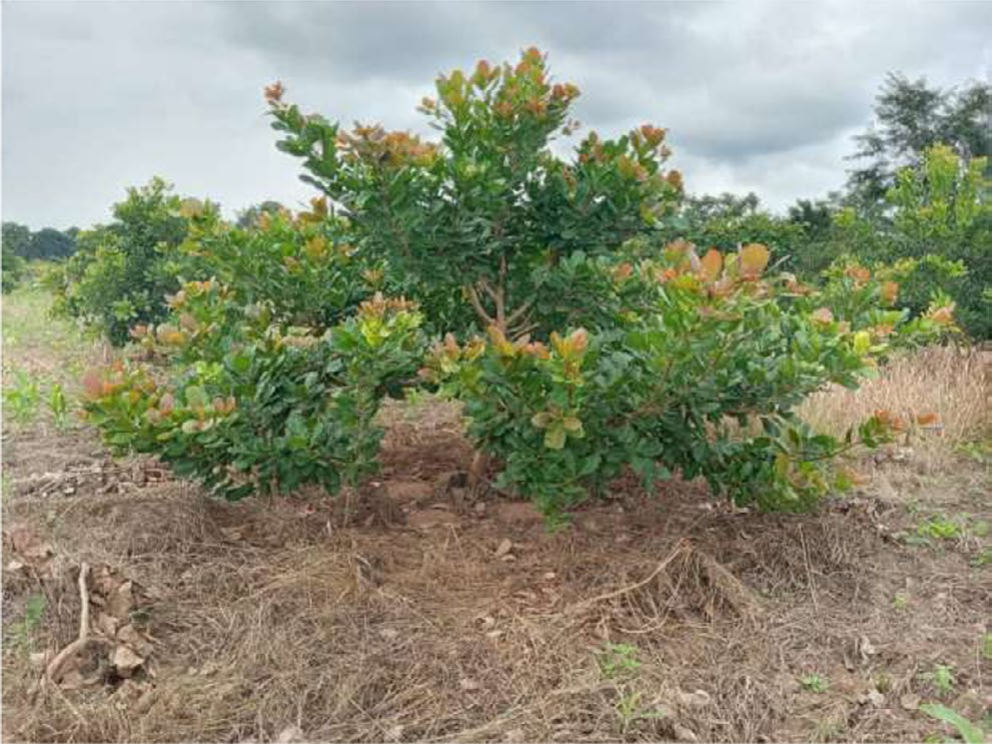
The Beninese cashew progeny SG287 × BE203 at two years after transplanting at Wenchi.

Among the 12 F_1_ progenies evaluated, significant differences (*p* < .05) were observed for all traits (Table 2). TCSAv values of the progenies ranged from 51.0 in SG287 × A2 to 92.0 cm^2^ in SG266 × BE203, and the Beninese progeny SG266 × BE203 was significantly vigorous compared to all the other progenies. On the other hand, TCSAr varied from 11,767 in SG266 × SG273 to 22,057 cm^2^ in SG266 × BE203. Similarly, the Beninese progeny SG266 × BE203 was highly vigorous; this was, however, closely followed by SG276 × SG273 and SG276 × A2 with TCSAr values of 18,897 and 15,865 cm^2^, respectively. For canopy spread in the north‐south (CSns) direction, a range of 2.8 in SG266 × SG273 to 4.6 m in SG287 × BE203 was recorded, whereas in east‐west (CSew) directions it varied from 1.9 in SG276 × BE203 to 3.8 m in SG266 × SG224, respectively. Again, the Beninese progeny SG287 × BE203 (Figure 1) gave significantly wider canopies in the north‐south direction while in the east‐west direction, the Ghanaian progeny SG266 × SG224 was outstanding.

**TABLE 2:** The mean growth and yield performance of 12 cashew progenies selected concurrently based on cropping efficiency and nut weight (*n* = 15) at Wenchi from 2012 to 2018

Progeny	TCSAv (cm^2^)	CSew (m)	CSns (m)	TCSAr (cm^2^)	Nut yield (kg/ha)	Cropping efficiency (g/cm^2^/year)	Rank	Nut weight (g/year)	Rank	† Average rank
SG266 × SG273	60abc	2.4ab	2.8a	11767a	490.1a	41.7ab	6	9.8de	2	8
SG266 × SG224	73bc	3.8d	3.5a	15428b	791.3cd	51.3bc	2	7.6bc	7	9
SG287 × BE203	71bc	3.5cd	4.6b	13959ab	939.4d	67.4c	1	7.4bc	8	9
SG266 × A2	51a	2.4ab	3.1a	15340b	577.6abc	37.5ab	9	10.5e	1	10
SG287 × SG273	69bc	3.64cd	3.6ab	12757ab	593.9abc	46.5ab	5	8.6cd	5	10
SG287 × A2	57ab	2.9abcd	3.4a	13117ab	516.7ab	40.1ab	7	8.5cd	6	13
SG276 × A2	67bc	2.7abc	3.5a	15865b	561.7ab	35.4ab	10	8.8cd	4	14
SG276 × SG224	68bc	3.7cd	3.7ab	14161ab	671.7abc	47.6ab	4	6.7ab	10	14
SG276 × BE203	65abc	1.9a	3.8ab	13167ab	669.8abc	50.9bc	3	6.4ab	11	14
SG276 × SG273	76c	3.0bcd	3.8ab	18897c	551.4ab	30.8a	12	9.7de	3	15
SG287 × SG224	70bc	3.3bcd	3.3a	13115ab	477.8a	38.4ab	8	7.4bc	8	16
SG266 × BE203	92d	3.3bcd	3.8ab	22057d	732.2bcd	33.6ab	11	5.9a	12	23
CV (%)	12.2	17.4	16.0	11.7	19.4	13.0		9.0		

Abbreviation: CSns: Canopy spread in the north‐south direction, CSew: Canopy Spread in the east‐west direction, TCSAr: Trunk cross‐sectional area at the vegetative stage, TCSAr: Trunk cross‐sectional area at the reproductive stage, CV: Coefficient of variation. Means followed by the same letter are not significantly (*p* ≤ .05) different based of p value for multiple comparison using Duncan multiple range test † Average rank across cropping efficiency and nut weight traits.

**FIGURE 2:**
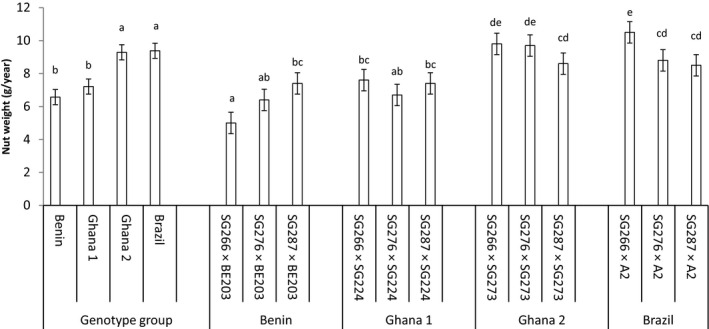
The mean nut weight of four genotype groups estimated from the combined means of 3 progenies planted at 15 trees per plot in three replications. Bars over the mean indicate ± standard error. Significant at *p* ≤ .05. Means followed by the same letters are not significantly (*p* ≤ .05). different based on the adjusted *p* ‐ value for multiple comparisons according to Duncan's multiple range test.

**FIGURE 3:**
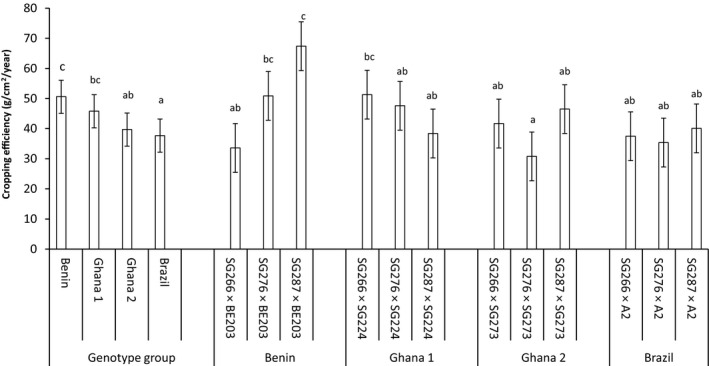
The mean cropping efficiency of four genotype groups estimated from the combined means of 3 progenies planted at 15 trees per plot in three replications. Bars over the mean indicate ± standard error. Means followed by the same letters are not significantly (*p* ≤ .05). different based on the adjusted *p* ‐ value for multiple comparisons according to Duncan's multiple range test

Nut yield among the progenies also ranged from 477.8 in SG287 × SG224 to 939.4 kg/ha in SG287 × BE203. Once more, the superior nut yield performance showed by SG287 × BE203 was closely followed by SG266 × BE203 and SG266 × SG224 giving nut yields of 791.3 and 732.2 kg/ha respectively. On the other hand, nut weight performance among the progenies ranged from 5.9 in SG266 × BE203 to 10.5g/year in SG266 × A2 and the superior performance of the Brazilian progeny SG266 × A2 was closely followed by two Ghanaian progenies SG266 × SG273 and SG276 × SG273 with nut weights of 9.8 and 9.7 g/year, respectively. For this trait, the Brazilian and one standard variety (Ghana 1 progenies) were comparable but superior to the Beninese and Ghana 2 progenies (Figure 2). Cropping efficiency performance also varied from 30.8 in SG276 × SG273 to 67.4 g/cm^2^/yr in SG287 × BE203 and again, the Beninese progeny SG287 × BE203 gave significantly high cropping efficiencies than all the progenies except SG266 × SG224 and SG276 × BE203 which gave mean cropping efficiencies of 51.3 and 50.9 g/cm^2^/year, respectively. In comparing the genotype groups, the Brazilian and the Beninese progenies were comparable to the Ghanaian genotype groups 1 and 2 for cropping efficiency and nut weight, respectively (Figures 2 and 3). However, the Beninese progenies were superior to the Brazilian progenies for cropping efficiency while for nut weight, the Brazilian progenies were superior to the Beninese progenies.

Overall, based on nut weight and cropping efficiency performance (obtained using a scale of 1–12 to score clones for each trait after which the total sum of scores were ranked), SG287 × BE203, SG287 × SG273, SG287 × A2, SG266 × A2, SG266 × SG273, and SG266 × SG224 were the top six outstanding progenies (Table 2).

### 3.3 Combining ability

Analysis of combining ability showed positive significant GCA effects for vigor (TCSAv), nut yield, canopy spread (CSns), and cropping efficiency for the Beninese male parent BE203.(Table 3) This indicates that it is a promising male parent for future breeding programs. However, it contributed negatively toward GCA effects for nut weight, indicating it is unsuitability as a male parent for high nut weight production. Meanwhile, the Brazilian (A2) and the Ghanaian (SG273) clone contributed positively toward GCA effects for nut weight indicating their appropriateness as a parent for producing hybrids with high nut weight. None of the female parents had a significant GCA effects for any trait except TCSAr where clones SG266 and SG278 showed significant positive and negative GCA effects, respectively. The negative GCA effects of SG287 for TCSAr indicate its suitability to reduce trunk girth at the reproductive stage, whereas the positive GCA effects showed by clone SG266 indicates its unsuitability as a parent for trunk girth reduction at the reproductive stage.

**TABLE 3:** Variance components from the performance of 12 cashew progenies derived from a factorial mating of three females and four males at Wenchi (*n* = 15) from 2012 to 2018

Variance components	TCSAv	TCSAr	CSns	CSew	Cropping efficiency	Nut yield	Nut weight
Male Variance	27.0	0.0	0.071	0.051	1.27	8,016.8	1.8
Female Variance	0.0	265272.4	0.034	0.000	0.0	0.0	0.0
Male x Female Variance	61.6	7302677.8	0.016	0.207	67.4	7,486.8	0.4
Additive Variance	335.0	29982412.1	0.394	0.995	273.9	56,184	7.7
Dominance Variance	246.6	29210711.4	0.062	0.828	269.7	29,947.4	1.7
Environmental Variance	23.0	1026997.9	0.108	0.094	32.9	4,683.8	0.2
Broad Heritability	0.96	0.98	0.81	0.95	0.94	0.95	0.98
Narrow Heritability	0.55	0.50	0.70	0.52	0.47	0.62	0.80

Abbreviations: CSns: Canopy spread in the north‐south direction, CSew: Canopy Spread in the east‐west direction, TCSAr: Trunk cross‐sectional area at the vegetative stage and TCSAr: Trunk cross‐sectional area at the reproductive stage.

### 3.4 Heritability estimates and correlation among traits

The narrow‐sense heritability estimates for TCSA at the vegetative and reproductive stages and canopy spread in the east‐west and north‐south, nut yield, cropping efficiency, and nut weight were 0.55, 0.50, 0.52, 0.70, 0.62, 0.47, and 0.80, respectively (Table 4). The genotypic (*r*g) and phenotypic (*r*p) correlation estimates among traits indicated that cropping efficiency had significant correlation with canopy spread in the north‐south direction (*r*_g_ = 0.98, *p* < .001; *r*_p_ = 0.56, *p* < .05) and nut yield (*r*_g_ = 0.70, *p* < .05; *r*_p_ = 0.74, *p* < .05 ). However, no significant correlation with vigor (expressed as increase in trunk cross‐sectional area, TCSA) at the vegetative (*r*_g_ = −0.23, *r*_p_ = −0.24) and reproductive (*r*_g_ = −0.51; *r*_p_ = −0.47) stages were observed, respectively. Additionally, nut weight negatively correlated with nut yield (*r*_g_ = −0.64, *p* < .05; *r*_p_ = −0.55^ns^ ) at the genotypic level (Table 5).

**TABLE 4:** General combining ability for trunk cross‐sectional area, canopy spread, cropping efficiency, nut yield, and nut weight of 12 cashew progenies derived from a factorial mating design of three females and four males at Wenchi (*n* = 15), Ghana

Pedigree	TCSAv	TCSAr	CSns (m)	CSew(m)	Cropping efficiency (g/cm^2^/year)	Nut yield (kg/ha/year)	Nut weight (g)
Male parents							
A2	− 4.890*	−0.003	−0.153	−0.127	−0.212	−52.550	1.056*
BE203	3.816*	0.001	0.310*	−0.048	0.270*	99.180*	−1.384*
SG224	1.102	0.001	−0.035	0.183*	0.085	10.490	−0.811
SG273	− 0.029	0.001	−0.122	−0.008	−0.137	−57.110	1.139*
SE 0.05	1.701	0.004	0.150	0.080	0.130	25.200	0.430
Female parents							
SG266	−0.001	133.192*	−0.154	0.001	0.001	0.001	−0.003
SG276	−0.002	62.511	0.063	0.001	−0.002	0.002	0.001
SG287	0.003	−195.704*	0.090	−0.002	0.001	−0.003	0.002
SE 0.05	0.003	65.100	0.130	0.003	0.009	0.001	0.004

* Estimates significantly (*p* ≤ .05) different from zero.

**TABLE 5:** Genotypic and phenotypic correlation coefficients for trunk cross‐sectional area, canopy spread, nut yield, cropping efficiency, and nut weight among 12 cashew progenies evaluated (*n* = 15) at Wenchi from 2012 to 2018

Traits		TCSAv (cm^2^)	CSns (m)	CSew (m)	Nut yield (kg/ha)	TCSAr (cm^2^)	Nut weight (g)
CSns (m)	Genotypic	0.74^**^					
	Phenotypic	0.38					
CSew (m)	Genotypic	0.56	0.42				
	Phenotypic	0.41	0.32				
Nut yield (kg/ha)	Genotypic	0.40	0.98^***^	0.43			
	Phenotypic	0.41	0.79^**^	0.40			
TCSAr (cm^2^)	Genotypic	0.87^**^	0.20	0.23	0.32		
	Phenotypic	0.88^**^	0.28	0.21	0.24		
Nut weight (g)	Genotypic	−0.82^**^	−0.89^**^	−0.44	−0.64^*^	−0.25	
	Phenotypic	−0.49	−0.46	−0.21	−0.55	−0.26	
Cropping efficiency (g/cm^2^/year)	Genotypic	−0.23	0.98^***^	0.27	0.70^*^	−0.48	−0.44
	Phenotypic	−0.24	0.56^*^	0.20	0.74^*^	−0.47	−0.31

Note

^*^, ^**^, ^***^ Significantly different at *p* ≤ .05, *p* ≤ .01, and *p* ≤ .001, respectively.

Abbreviations: CSns: Canopy spread in the north‐south direction, CSew: Canopy Spread in the east‐west direction, TCSAv: Trunk cross‐sectional area at the vegetative stage, TCSAr: Trunk cross‐sectional area at the reproductive stage.

## 4 DISCUSSION

The productivity of cashew over the years has been constrained by low nut yield with smaller nut weights (Adu‐Gyamfi, Dadzie, et al., 2019; Rabany et al., 2015). This has been attributed to the narrow genetic base of commercially cultivated varieties (Archak et al., 2003) in addition to the change in production environment (long dry spells, erratic rainfall patterns, and extreme temperature) (Bello et al., 2017). In tree crop breeding, improving the cropping efficiency of existing varieties has been considered to be a viable strategy to improve productivity through high‐density planting (Daymond et al., 2002; Padi, Ofori, & Akpertey, 2017). The emphasis of the present study was to identify clones with good combining ability for reduced tree size and high nut weight in the early years of bearing.

Glendinning (1960) has emphasized that selecting for vigorous tree crop varieties at the vegetative stage may lead to high yielding trees that are too large to manage. This has been counteracted by the use of cropping efficiency which is an index that integrates yield with vegetative growth. Cropping efficiency has been found to be a better indicator of productivity than yield itself (Daymond et al., 2002). Many tree crop improvement programs have reported significant GCA and SCA effects on vigor, bean weight, and cropping efficiency (Daymond et al., 2002; Ofori, et al., 2014; Padi et al., 2017).

In the present study, significant GCA and SCA effects existed for most of the tested traits including vigor, cropping efficiency, and nut weight, which implied that both additive and non‐additive allelic effects were important in controlling these traits as stated by Griffing (1956). However, the much greater than unity ratio of GCA:SCA variances suggest the predominant role of additive gene effects in controlling most traits. Our results are consistent with what has been found in cashew (Cavalcanti et al., 2000; Cavalcanti et al., 2007; Wunnachit et al., 1992) and other tree crops such as cocoa (Pang, 2006; Tan, 1990) and almonds (García et al., 1994). Nevertheless, reports on GCA effects on cropping efficiency and nut weight in cashew are rare. The positive GCA effects of the male parent BE203 for cropping efficiency, nut yield, canopy spread, TCSAv and A2 together with SG273 for nut weight implied that they could possess favorable alleles and contribute to the improvement of these traits in an additive fashion, respectively. The significant positive GCA by the Brazilian clone A2 for nut weight in our study agrees with the reports of Aliyu and Awopetu (2011) who emphasized that the Brazilian Dwarf germplasm accessions were storehouses of genes for larger "jumbo" nut weights. Nevertheless, GCA for A2 and BE203 were negative for TCSAv and nut weight which suggest that they have limited potential as male parents in the improvement of these traits, respectively. Interestingly, for any given trait measured in this study, at least one male parent showed a significant GCA effect and this observation demonstrate the amenability of all the tested traits to genetic manipulation through conventional breeding. But, surprisingly, none of the female parents showed significant GCA effect on any given trait except TCSAr where a significant negative GCA effect was observed with SG287, whereas a positive effect was observed for SG266. This suggests that while SG287 partitioned small amount of assimilates to reproductive growth, SG266 partitioned large amounts of assimilates to vegetative growth. Similar observations in cocoa (*Theobroma cacao* L.) has been reported (Padi, Ofori, & Akpertey, 2017). Enhancing this trait in the future would require a wide array of parental clones with good combining abilities.

The greater narrow‐sense (˃45%) heritability estimates observed for all the tested traits implied that environmental influence was minimal and considerable selection responses can be expected. In Brazil, Cavalcanti, de Resende, et al. (2007) reported similarly high narrow‐sense heritability estimates for nut weight (63.2%) but low heritability estimate for nut yield (21%). However, Masawe et al. (1999) reported low narrow‐sense heritability range for nut yield (3%–40%) and trunk cross‐sectional area (14.7%–38.9%) in Tanzania. In the present study, high heritabilities for nut yield (62%) and trunk cross‐sectional area (55%) were observed. The observed differences in the magnitude of heritability estimates for these traits have been attributed to genetic constitutions of varieties tested, environmental conditions, and genotype × environment interactions effects (Crossa et al., 1990).

With the exception of the non‐significant correlation between TCSA and cropping efficiency, the genetic correlations observed in our study are consistent with what has been found in other tree crops. Genetic correlations were reported between cropping efficiency and yield (Daymond et al., 2002; Padi et al., 2012) and between yield and canopy spread (Aliyu, 2006; Masawe et al., 1999). The non‐significant correlation between TCSA at the reproductive stage and cropping efficiency and the significant correlation between canopy spread in the north‐south direction and cropping efficiency in this study indicates the importance of breeding for wider canopies rather than focusing on reduced TCSA at the reproductive stage to achieve high cropping efficiency. As the GCA for BE203 was also significant and positive for canopy spread (CSns) and TCSA at the reproductive stage, it would be an excellent resource to be deployed in future breeding programs to produce cashew varieties with high cropping efficiencies with better field establishment as high vigor (TCSAv) in tree crops is associated with high seedling survival rates especially under marginal field conditions (Ofori et al., 2015). This finding supports the report of Adu‐Gyamfi, Abu Dadzie, et al. (2019) who indicated that the Benin clones could possibly possess alleles that ensures higher water and nutrient‐use efficiency with better tolerance to high temperature stress. Nevertheless, in cashew breeding, varieties with compact canopies are preferred as it affords the opportunity to increase yield through increasing plant densities on per hectare basis (Aliyu & Awopetu, 2007). Therefore, further traits improvement would require a wide array of parental clones with good combining abilities.

On the other hand, the negative significant correlation between nut weight and nut yield and canopy spread indicates that the lower the nut weight, the higher the corresponding nut yield and the wider the canopies. Both positive and negative correlations between nut yield and nut weight has been reported in cashew (Aliyu, 2006; Sena et al., 1994). The conflicting reports on these associations have been attributed to differences in the advanced stage of the population utilized and the nature of the previous selection method employed in the previous yield improvement program (Paikra, 2016; Sundararaju et al., 2006). Madeni (2016) has therefore indicated that their use as a selection criterion in the breeding program under all ecologies has therefore demonstrated significant indirect effects on yield through the number of nuts per tree without any adverse compensation effects. The significant and positive GCA for A2 and SG273 for nut weight suggest that they could constitute a suitable genetic resource for future breeding programs that aim to develop cashew varieties with higher nut weights. To attract better prices for premium kernels, nut weights in the range of ~9–12 g have been recommended for farmers (Aliyu & Awopetu, 2011). The ideal variety in this study is therefore considered as one that combine high cropping efficiency with high nut weights. A comprehensive evaluation of progeny performance based on multiple traits has been found to be more stable and accurate. Ofori, et al. (2014) selected best and worst cocoa progenies concurrently based on overall trait performance using a scale of 1–24 to score for each trait after which the total scores were ranked. Based on cropping efficiency and nut weight the following progenies, SG287 × BE203, SG287 × SG273, SG276 × SG224, SG266 × A2, SG266 × SG273, and SG276 × BE203 are likely to be of value. These progenies developed from parents with low × high GCA values might be attributable to complementation of low and high combining loci as reported in other studies by Raut et al. (2017).

The potential of the identified progenies could further be validated under different high planting densities in multi‐location tests under farmers’ production conditions across the cashew production belts in the West African cashew producing countries to determine those best adapted to specific agro‐ecological regions.

## 5 CONCLUSION

Our study demonstrated that the potential vigor of cashew tree is under genetic control and the Brazilian (A), Beninese (BE), and Ghanaian (SG) clones had different merits as potential parents for TCSAv, TCSAr, cropping efficiency, and nut weight. The comparatively higher nut yields in the range of 477.8–939.4 kg/ha and cropping efficiency varying from 30.8 to 67.4 g/cm^2^/year and nut weights from 5.9 to 10.5g underscores the importance of broadening the genetic base for production. The Ghanaian progenies (standard variety) were comparable to the Brazilian and Beninese progenies for nut weight and cropping efficiency, respectively. However, based on these two traits, six progenies, SG287 × BE203, SG287 × SG273, SG276 × SG224, SG266 × A2, SG266 × SG273, and SG276 × BE203, were outstanding, revealing the ability to combine targeted traits into a single background. Additive effects seemed to play a dominant role in the inheritance of most traits and breeding should therefore focus on cashew parents that are general combiners. Among the males, BE203 showed significant GCA effects for cropping efficiency, nut yield, TCSA at the vegetative stage, and canopy spread in the north‐south direction, whereas A2 and SG273 demonstrated significant positive effects on nut weight. Among the females, SG266 and SG278 showed positive and negative GCA effect on TCSA at reproductive stage respectively. Cropping efficiency correlated strongly (*r*_g_ = 0.98, *p* < .001) with canopy spread in the north‐south direction at the genotypic level. The identified clones constitute a suitable genetic resource pool for improving cashew productivity.

## CONFLICT OF INTEREST

The authors declare that there is no conflict of interest.

## AUTHORS’ CONTRIBUTION

PKKAG and MB conceived the manuscript. PKKAG collected and analyzed the data, wrote the manuscript. AA, AO, and FP all assisted with data collection and provided significant editorial and analytical advice.

## FUNDING INFORMATION

The authors acknowledge the support of the Deutsche Gesellschaft für Internationale Zusammenarbeit GmbH (GIZ) with funding through the African Cashew Initiative (ACi) and the Cocoa Research Institute of Ghana (CRIG).

## Supporting information

Table S1
