# Supplementary material for: Genotypic characterization of cashew (Anacardium occidentale L.) clones using agro‐morphological traits
Source: Plant Environ Interact. 2020 Dec 2;1(3):196–206. doi: 10.1002/pei3.10034 (PMC10168051; doi:10.1002/pei3.10034)
Supplement: Supplementary file 1 — Table S1 [file PEI3-1-196-s001.doc]

Table S1. Soil characteristics of experimental site at Wenchi, Ghana.

| Parameters | Mean (± standard error), n = 16 | Recommended soils † |
| --- | --- | --- |
| Soil pH | 5.13 ± 0.371 | 5.2 - 7.5 |
| Organic Carbon (%) | 1.16 ± 0.004 | 2.0 |
| Total Nitrogen (%) | 0.11 ± 0.008 | 0.10 |
| Available Phosphorus (ppm) | 14.2 ± 1.861 | 10.0 |
| Exchangeable potassium (cmol/kg) | 0.23 ± 0.032 | 0.20 |
| Exchangeable magnesium (cmol/kg) | 1.48 ± 0.021 | 0.80 |
| Soil class | Lithosol |  |

† Dedzoe et (2001). Characteristics of recommended cashew growing soils from analyses of soils from different agro-ecologies in Ghana
